# Investigation of the physicochemical factors affecting the in vitro digestion and glycemic indices of indigenous indica rice cultivars

**DOI:** 10.1038/s41598-025-85660-5

**Published:** 2025-01-17

**Authors:** Indira Govindaraju, Anusha R. Das, Ishita Chakraborty, Sib Sankar Mal, Bhaswati Sarmah, Vishwa Jyoti Baruah, Nirmal Mazumder

**Affiliations:** 1 Department of Biophysics, Manipal School of Life Sciences, Manipal Academy of Higher Education, Manipal, Karnataka 576104 India; 2 Materials and Catalysis Lab, Department of Chemistry, National Institute of Technology Karnataka, Surathkal, Karnataka 575025 India; 3 Department of Plant Breeding and Genetics, Assam Agricultural University, Jorhat, Assam 785001 India; 4 Centre for Biotechnology and Bioinformatics, Dibrugarh University, Assam, 786004 India

**Keywords:** In vitro digestion, Glycemic index, Amylose content, Resistant starch, Swelling power, Water uptake, Cooking qualities, Biophysics, Biotechnology

## Abstract

Rice *(Oryza sativa*) is a vital food crop and staple diet for most of the world’s population. Poor dietary choices have had a significant role in the development of type-2 diabetes in the population that relies on rice and rice-starch-based foods. Hence, our study investigated the in vitro digestion and glycemic indices of certain indigenous rice cultivars and the factors influencing these indices. Cooking properties of rice cultivars were estimated. Further, biochemical investgations such as amylose content, resistant starch content were estimated using iodine-blue complex method and megazyme kit respectively. The in vitro glycemic index was estimated using GOPOD method. The rice cultivars considered in our study were classified into low-, intermediate-, and high-amylose rice varieties. The rice cultivars were subjected to physicochemical characterization by using Fourier transform infrared (FTIR) spectroscopy and differential scanning calorimetry (DSC) techniques. FTIR spectral analysis revealed prominent bands at 3550-3200, 2927-2935, 1628-1650, 1420-1330, and 1300-1000 cm^−1^, which correspond to –OH groups, C=O, C=C, and C–OH stretches, and H–O–H and –CH bending vibrations, confirming the presence of starch, proteins, and lipids. Additionally, the FTIR ratio R(1047/1022) confirmed the ordered structure of the amylopectin. DSC analysis revealed variations in the gelatinization parameters, which signifies variations in the fine amylopectin structures and the degree of branching inside the starch granules. The percentage of resistant starch (RS) ranged from 0.50–2.6%. The swelling power (SP) of the rice flour ranged between 4.1 and 24.85 g/g. Furthermore, most of the rice cultivars are classified as having a high glycemic index (GI) based on the estimated in vitro GI (eGI), which varies from 73.74–90.88. The cooking properties of these materials were also investigated. Because the amylose content is one of the key factors for determining the cooking, eating, and digestibility properties of rice, we investigated the relationships between the amylose content and other biochemical characteristics of rice cultivars. The SP and GI were negatively correlated with the amylose content, whereas the RS had a positive relationship. The findings of our study can be beneficial in illustrating the nutritional profile and factors affecting the digestibility of traditional rice cultivars which will promote their consumption, cultivation, and contributes to future food security.

## Introduction

Rice *(Oryza sativa*) is one of the world’s most important food crops, and it is a staple diet for almost half of the world’s population. Rice production and consumption are the highest among the Asian population, especially among Indians^1^. Rice production in India accounts for 20% of the global rice production. The states of Karnataka (191,791 km^2^, latitudes 11.5° N and 18.5° N and longitudes 74° E and 78.5° E)^2^ and Assam (tropical latitudes (24° 08′ N to 27° 59′ N) and eastern longitudes (89° 42′ E to 96° 01′ E)^3^ are naturally endowed with a wide variety of flora and different climatic conditions that support the cultivation of novel, indigenous rice cultivars. Furthermore, Karnataka has a tropical climate with a temperature of approximately 28 °C. and lies in the X and XII agroclimatic zones, and the annual rainfall is 1248 mm^2^. There are different types of soils throughout the state that influence agricultural cropping patterns. However, Assam is characterized by a tropical temperature of 10.4–32.3 °C, significant rainfall, and high humidity. Hence, two different zones of agro-climatic conditions support the growth of a wide variety of indigenous, nutrient-rich rice cultivars that vary in their nutritional properties and rate of digestion. Traditional rice varieties are predominantly cultivated and consumed around the world, and farmers are interested in these rice cultivars due to their superior grain quality. Since each cultivar demonstrates ecological adaptability, they are very important genetic reserves due to their time-tested features^4^. Among such traditional rice cultivars, some of the most prominent rice cultivars cultivated in Karnataka are Rajamudi, Rathnachoodi, Gandha Saale, and Jeerige Sanna. Joha, Bau, Sali, and Chakaw are the most well-known in India’s Northeast Region. The Rajamudi rice cultivar is an exotic and prominent variety that was once highly desired for use in royal kitchens and courtly cuisine. Rajamudi rice varieties were specifically grown for the kings of the Mysore region in Karnataka. It has two varieties based on the color of the rice kernel, Bili (white) and Kempu (red) Rajamudi red rice varieties are often used by ayurvedic practitioners to treat various diseases, such as hypertension and digestive issues. In particular, red rice porridge is fed to people who are suffering from fever because it aids in maintaining optimal body temperature. Gandha (fragrant/aroma) Saale, Joha and Jeerige Sanna are aromatic rice varieties that are used on special occasions due to their fragrance and finest cooking properties. Furthermore, traditional aromatic rice varieties are used to treat anemia since they possess high iron and zinc contents and thus increase the bioavailability of iron. Bananthi (lactating women) and Akki (rice) are normally fed to lactating women because they are said to provide great stamina and promote lactation. Many traditional rice varieties are often cooked and consumed as whole grain, and often, they are described as functional foods due to their nutraceutical properties. However, some of these products are efficiently processed into rice-based food products such as rice noodles, rice cakes, rice flakes, puffed rice and other rice and rice starch-based delicacies that are popular among Asians. Since dietary carbohydrates are the primary source of energy for most people, understanding the quality of carbohydrates is crucial for the dietary management of metabolic diseases such as type 2 diabetes and obesity. Poor diet choices are the key contributors to the development of type 2 diabetes and obesity. In particular, rice and other starch-based foods are consumed. Determining the glycemic index and resistant starch content is an important approach for assessing the quality of carbohydrate-rich foods. The consumption of low-glycemia-indexed foods and high-resistance starch foods is associated with many health benefits, such as improved insulin sensitivity, improved digestive health, decreased risk of type 2 diabetes, and reduced hemoglobin A1c (HbA1c)^5–7^. Various studies have reported that a wide array of traditional red rice cultivars are associated with medicinal and nutritional properties, which in turn provide many health benefits for people after consumption. Additionally, local communities have several other perspectives about these traditional rice cultivars in addition to being a staple diet. Hence, in our study, we determined the in vitro digestibility, glycemic index, and resistant starch content of unpolished traditional rice cultivars that are endemic to the states of Karnataka and Assam in India. Furthermore, we characterized their cooking properties, gelatinization parameters using differential scanning calorimetry (DSC), and alterations in their chemical structure owing to differences in digestibility using Fourier transform infrared (FTIR) spectroscopy. The main aim of this study was to compare the in vitro digestibility of traditional rice cultivars grown in the region of Karnataka and Assam and to establish the relationships among the amylose content, glycemic index, resistant starch content and digestibility. Currently, in addition to being a staple diet, these traditional rice types are considered from several other perspectives. Hence, the findings of our systematic study will be beneficial for demonstrating the nutritional profile and factors affecting the digestibility of traditional rice cultivars from Karnataka and Assam, which will help to support the competitiveness of the global market and promote the consumption and cultivation of traditional rice varieties.

## Materials and methods

### Sample collection

The rice cultivars were collected from the local farmers of Karnataka (Bananthi akki, Gandha Saale, Bili Rajamudi, Betta Sanna, Salem Sanna, Tanu, Bili Muguthi, Kempu Rajamudi, Kage Thali, Kaveri) and Assam (Neuli, Thangam Champa, Til Bora, Kununkuruvai, Jhum Beji, Chakaw, Aad Bau, Kutkuti Sali, Basmathi, Pare) regions of India. The samples were dehusked to obtain rice grains. A 10 g of rice grains were finely ground using a mixer (750 Watts, Prestige Delight, India) and sieved through through a 1.0-mm sieve screen to obtain rice flour. It is stored in air-tight container till further analysis.

### Physical characteristics of the rice grains

The phenotypical characteristics of dehusked rice grains are characterized. The physical parameters, such as the length, width, and thickness of the rice grains, were measured using digital Vernier calipers.

### Determination of cooking time, water uptake ratio, grain elongation ratio, and volume expansion ratio

#### Cooking time

The cooking time was estimated by the open vessel cooking method. Rice kernels (5 g) were cooked in 100 mL of distilled water. Meanwhile, a countdown clock was started. For every 5 min, a few rice kernels were removed from the cooking vessel and sandwiched between glass slides to check for the absence of white cores in the rice kernels to ensure complete cooking. The countdown clock is stopped once the cooking is completed, and the final time is recorded as the established cooking time^8,9^.

#### Water uptake (WU)

The minimum quantity of water taken by rice kernels during the process of cooking is referred to as water uptake. Two grams of rice was weighed and cooked in 10 mL of water until the rice was completely cooked. Individual rice kernels were removed and sandwiched between glass slides so that there was no white core, which ensures complete cooking. The cooked rice was then drained, and the excess water was measured. The amount of water absorbed is calculated by subtracting the amount of drained water from the amount of initial water added. Then, the water uptake of the different rice cultivars was expressed as 100 g of rice^8^.

#### Grain elongation ratio (ER) and volume expansion ratio (VER)

The initial grain length (L0) was measured using digital Vernier calipers before cooking, and the final grain length (L1) was recorded after cooking^10^. The grain elongation ratio was calculated as the ratio of the length of cooked rice to the length of uncooked rice kernels, and the volume expansion ratio was measured by the water displacement method and expressed as the ratio of the volume of cooked rice grains to the volume of raw rice grains^8^.

### Fourier transform infrared (FTIR) spectroscopy of the rice varieties

FTIR spectroscopy was used to examine alterations in the chemical composition of rice flour from different varieties of rice. The rice flour samples were blended with KBr and mounted onto a potassium bromide (KBr) pellet holder. The spectra of the mounted rice flour samples were recorded using a Bruker alpha FTIR spectrometer (Bruker alpha, Germany), and the samples were scanned from 4000 to 500 cm^−1^. The spectra obtained from different rice flour samples were analyzed by monitoring the presence and absence of functional groups, chemical bonds, bond stretching, and molecular vibrations.

### Differential scanning calorimetry (DSC) of rice varieties

The gelatinization parameters were determined using a differential scanning calorimeter (Shimadzu’s DSC60). The sample preparation was performed by coating approximately 3–4 mg of rice flour sample (~ 10% moisture) with 2µL distilled water in an aluminum pan. The pan was manually crimped using a sample-encapsulating press (Shimadzu’s DSC60), heated at specific temperatures between 30 and 110 °C at intervals of 10 °C and monitored for DSC curves. The onset, peak, and endset temperatures and the transition enthalpy (ΔH) were recorded. The DSC curves were analyzed using TA-60WS software.

### Determination of amylose content in the rice varieties

To determine the amylose content of the starch granules, 100 mg of rice flour was weighed into a 100 mL volumetric flask, and 1 mL of 99% ethanol and 9 mL of 1 N NaOH solution were added to the flask. The gelatinization of starch was carried out by heating it in boiling water for 10 min. The solution was then cooled and made up to the mark in the flask with distilled water. Five milliliters of the sample solution was transferred to another 100 mL standard flask. After that, 1 mL of 1 M acetic acid and 2 mL of iodine solution were added, the solution was diluted with distilled water, and the absorbance was measured using a UV‒Vis spectrophotometer at 620 nm. The amylose content was calculated using the standard graph of potato amylose^11,12^.

### Determination of the swelling power of the rice varieties

One gram of rice flour was weighed and transferred to 40 mL of distilled water. The mixture was incubated in a water bath at different temperatures ranging from 55 to 95 °C for approximately 30 min. All the tubes were cooled to room temperature and centrifuged (Thermo Scientific™ Sorvall™ ST 8 Centrifuge) at ×1000*g* for approximately 20 min. The resulting pellet was recovered, and the swelling power was calculated as the ratio of the wet sediment weight to the dry rice flour weight^13^.

### Determination of resistant starch (RS) and total starch (TS)

The RS content of the rice samples was estimated using a megazyme RS assay kit (Megazyme International Ireland Ltd., Bray, Ireland) according to the manufacturer’s protocol. One hundred milligrams of rice flour was digested with 4 mL of pancreatic α-amylase (10 mg/mL) in a 50 mL conical flask in the presence of amyloglucosidase (300 U/mL). The digestion mixture was incubated at 37 °C with continuous stirring at 200 rpm for 16 h. Then, 4 mL of absolute ethanol was gently added. The contents were then centrifuged at ×1500*g* for 10 min to separate the pellet and supernatant. Two milliliters of 50% ethanol was added to the pellet while vigorously stirring, and the volume was increased to 8 mL by adding 50% ethanol. The pellet was further centrifuged at ×1500*g* for 10 min. The supernatant was collected in separate tubes and processed to estimate the nonresistant (digestible) starch content. The centrifugation process was repeated three times to accumulate a total volume of 24 mL of the supernatant, and the remaining drops of the supernatant were removed by inverting the tubes on the absorbent paper. Two milliliters of 2 M KOH was used to resuspend the pellet with continuous stirring in an ice bath for approximately 20 min, followed by the addition of 8 mL of 1.2 M sodium acetate buffer (pH 3.8) along with 0.1 mL of amyloglucosidase (3300 U/mL). The tubes were vortexed well, incubated at 50 °C for 30 min and centrifuged at ×1500*g* for 10 min. Then, 0.1 mL of the supernatant was aliquoted into glass test tubes in triplicate, and 3 mL of glucose determining reagent, glucose oxidase peroxidase (GOPOD), was added to the tubes, which were then incubated at 50 °C for 20 min. The resulting pink complex was subjected to colorimetric absorbance at 510 nm against a blank, and the RS and TS concentrations were calculated according to the manufacturer’s formula.

### Determination of the in vitro glycemic index (GI) of the rice varieties

The estimated GI of the rice varieties was determined according to the method described by Goñi et al.^14^ Briefly, 3 g of boiled rice was put into an Erlenmeyer flask, to which 0.2 mL of pepsin (1 g/10 ml HCl-KCl buffer, pH – 1.5) was added. The reaction was accelerated by incubating the mixture at 40 °C for 1 h in a shaking water bath. After the end of the incubation period, the total volume of the solution was adjusted to 25 ml with Tris-Malaete buffer (pH 6.9), followed by the addition of 5 ml of porcine pancreatic amylase (2.6 U). The mixture was again incubated at 37 °C for approximately 3 h with continuous stirring. One-millilitre aliquots were taken from this solution every 30 min until the end of the incubation period. The aliquots taken were labeled for 0, 30, 60, 90, 120, 150, and 180 min and placed in a boiling water bath for approximately 5 min with intermittent shaking to decrease enzyme activity. One milliliter of 0.4 M sodium acetate buffer (pH 4.75) and 60 µL of amyloglucosidase were added to each aliquot and incubated at 60 °C for 45 min. Later, the total volume was adjusted to 100 mL with distilled water, and the amount of glucose released was measured with a glucose oxidase peroxidase assay.

The kinetics of starch hydrolysis followed pseudo-first-order kinetics C=C_∞_(1 − e^−kt^), where C, C_∞_, and k are the percentage of starch hydrolyzed at time ‘t’ (min), the equilibrium percentage of starch hydrolyzed at 180 min, and the kinetic constant, respectively. The hydrolysis index (HI) was calculated by dividing the area under the hydrolysis curve of each sample by the area of a reference material, in this case, white bread. The estimated GI (eGI) was calculated using the equation GI = 39.71 + (0.549HI)^14^.

### Statistical analysis

The mean and standard deviation of triplicate data from the experiments were calculated using Microsoft Excel 2019. Analysis of variance was calculated using GraphPad Prism 8.0.2 by applying Tukey’s test to determine the statistical significance of the values obtained. The results with *p* < 0.05 were considered to be significant. Pearson correlations (‘r’ values) among the amylose content, resistant starch content, and in vitro glycemic index were determined. An ‘r’ value between -1 and 0 indicates a negative correlation, whereas a value between 0 and 1 indicates a positive correlation.

## Results and discussion

### Phenotypic characterization and determination of cooking parameters

**Fig. 1:**
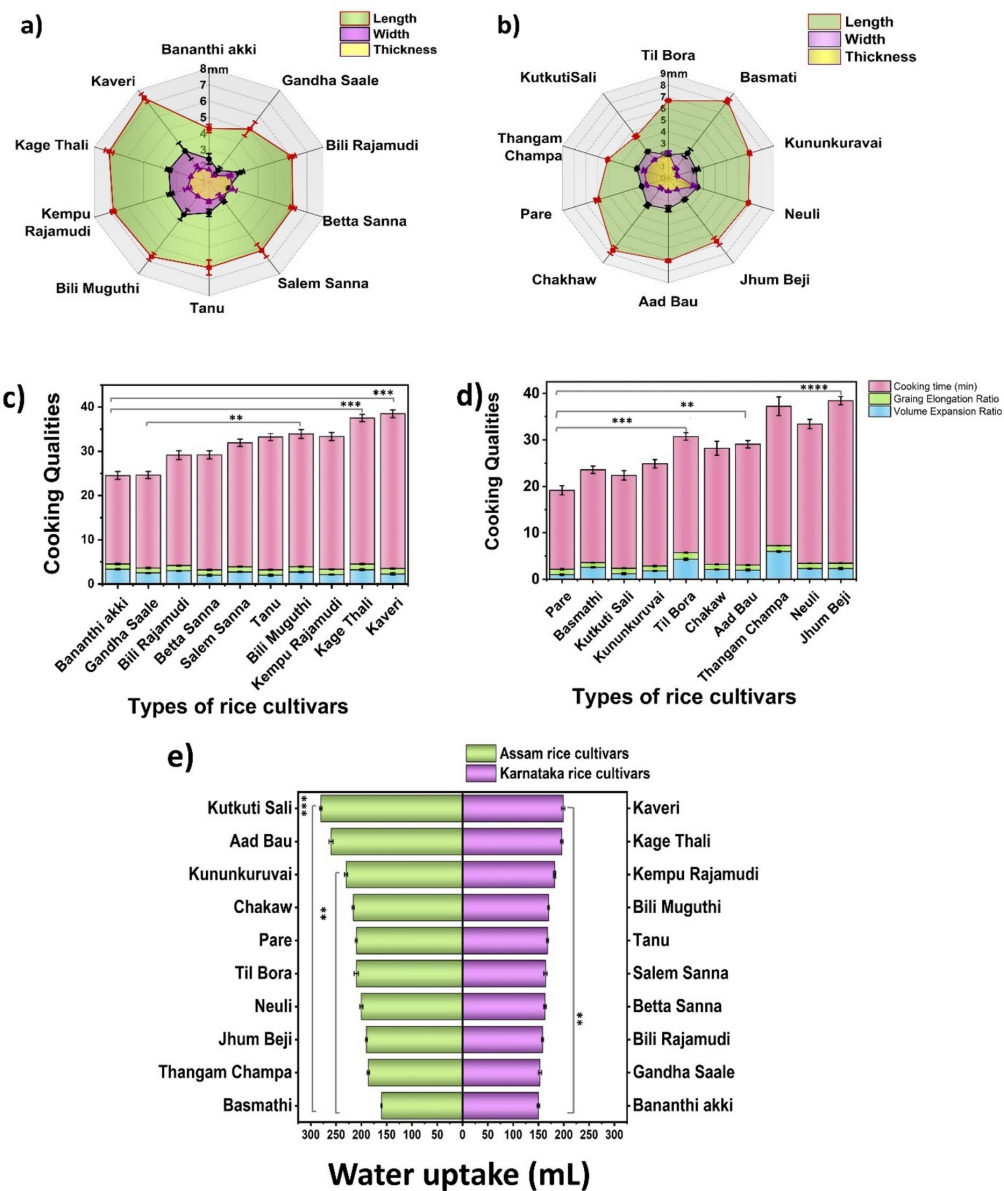
Graphical representation of the cooking parameters of (**a**) Karnataka (**b**) Assam rice cultivars, and (**c**) Bar graph showing the water uptake of rice cultivars (*** *p* = 0.019, ** *p* = 0.037).

Basmathi had the greatest elongation ratio (1.41 ± 0.12), followed by Thangam Champa (1.25 ± 0.17) and Pare (1.16 ± 0.11), as shown in Fig. 1. However, the minimum elongation ratio was observed in Til Bora (1.02 ± 0.12) and Chakaw (1.10 ± 0.14). The minimum cooking time of the rice varieties was determined when no longer the white core of the kernels was visible. For the Karnataka rice variety, Bili muguthi took the most time, i.e., 35 min, and Bananthi akki took the least time, 20 min, to cook, whereas for the Assam rice variety, Kununkuruvai took the most time, i.e., 35 min, and Aad Bau took the least time, 17 min, to cook. In the Karnataka rice variety, Kaveri consumed 199 mL of water, and Bananthi Akki consumed 150 mL of water, whereas in the Assam variety, Kutkuti Sali consumed 270 mL of water, and Basmathi consumed 160 mL of water for cooking. Kaveri had a maximum grain length of 7.39 ± 0.32 mm, and Bananthi Akki had the minimum grain length of 4.30 mm among the Karnataka varieties, whereas Basmathi had the maximum grain length (8.20 ± 0.22), and Til Bora had the minimum grain length (1.02 ± 0.051 cm) among the Assam varieties. The maximum volume expansion ratio was observed in Bananthi Akki (3.33 ± 0.15), and the minimum volume expansion ratio was 2 ± 0.21, which was observed in Tanu and Betta Sanna from the Karnataka rice variety, whereas in the case of the Assam rice variety, Jhum Beji had the maximum volume expansion ratio (4.33 ± 0.21) and minimum volume expansion ratio (1 ± 0.15). These cooking parameters are interconnected and interdependent, and they are affected by various factors, such as rice variety and genetics, cooking time and temperature, water composition, and environmental conditions during cultivation. The differences in water uptake behavior may be due to variations in the key genetic loci associated with water uptake in rice^15^. Furthermore, rice cultivars grown at different altitudes have been shown to possess various physical, chemical, and cooking attributes^16^, which supports the differences in the cooking characteristics of the Assam and Karnataka rice cultivars. Climate change-induced shifts in temperature patterns, rainfall distributions, and extreme weather events can have profound impacts on rice grain characteristics^17^.

### Fourier transform infrared (FTIR) spectroscopy of the rice cultivars

**Fig. 2:**
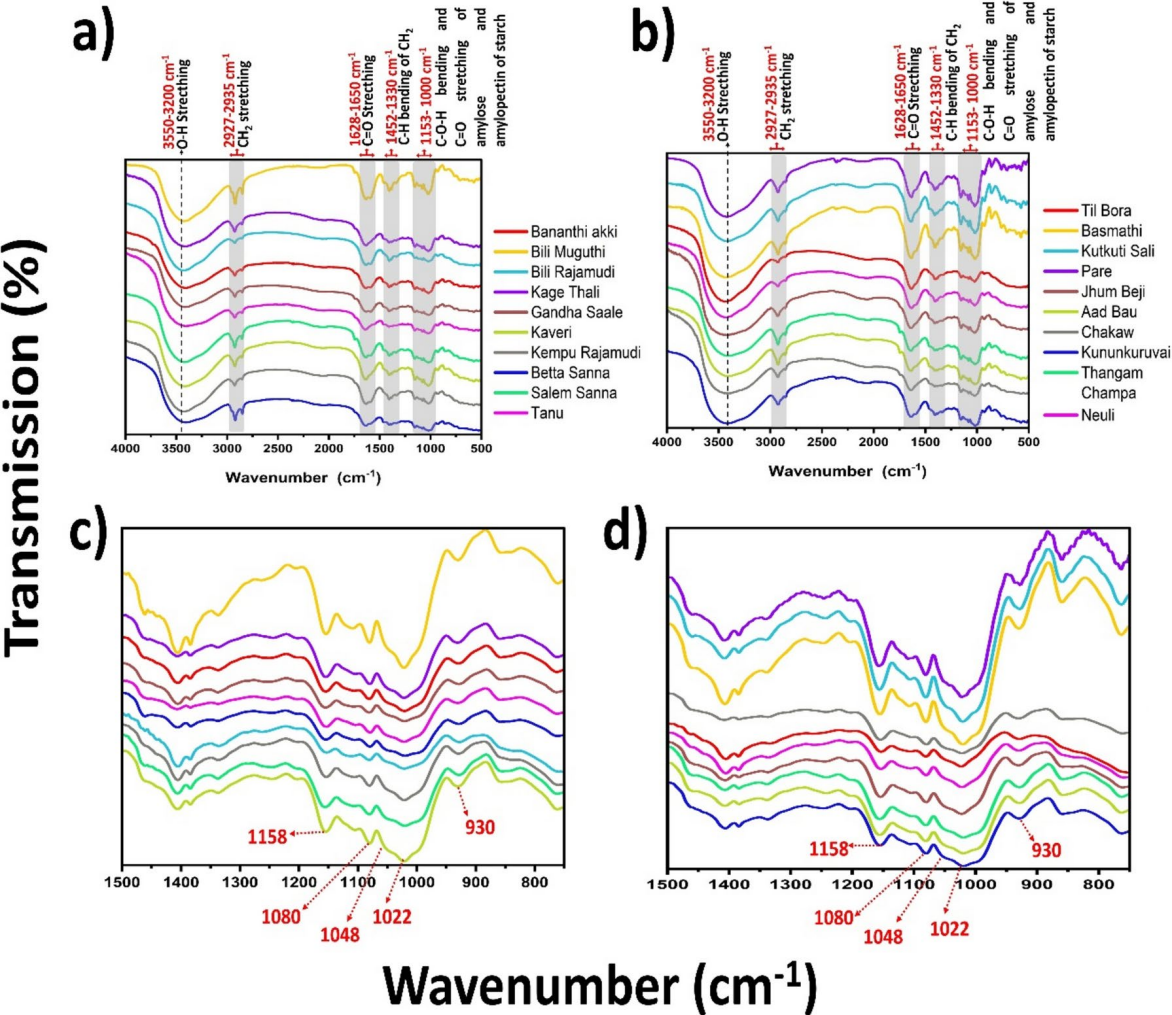
FTIR spectra of (**a**) & (**c**) Karnataka; (**b**) & (**d**) Assam rice cultivars; (**c**) & (**d**) fingerprint region of the FTIR spectra of the Karnataka and Assam rice cultivars, respectively.

The FTIR spectra of the Karnataka and Assam rice cultivars revealed multiple chemical bonds that are structurally positioned in the molecule. We scanned rice flours in the range of 4000–800 cm^−1^, and there was not much significant variation in the spectra of the Karnataka and Assam rice cultivars. This is presumably due to the similar structural architecture of the predominant starch polysaccharide, which is present in all the common rice varieties. Prominent peaks were found at 3550–3200 cm^−1^, 2927–2935 cm^−1^, 1628–1650 cm^−1^, 1420–1330 cm^−1^, and 1300–1000 cm^−1^ (Fig. 2a,b). The wide and broad FTIR bands at 3550–3200 cm^−1^ correspond to the -OH groups exhibiting stretching vibrations, which confirms that the polysaccharide component is the major moiety present in the rice flour. The weak and medium-length bands at 2927–2935 cm^−1^ and 1628–1650 cm^−1^, respectively, represent CH_2_ asymmetric or symmetric stretching due to the presence of nonstarch components such as lipids and proteins as well as the C=O stretch (1652 cm^−1^) Cis C=C (1654 cm^−1^) (Table 1), which indicates the presence of starch-bound proteins. This confirms that these moieties influence the digestion of rice cultivars and thus the estimation of the GI. Although proteins and lipids are present in minor fractions, they contribute to the structural integrity of starch granules and affect the thermal and rheological properties of starch. In addition, they influence the texture, cooking characteristics, and sensory attributes of cooked rice and contribute to its overall nutritional quality^18^, ^19^ These bands are also attributed to the H–O–H bending of the bound water molecules, which represents the moisture content of the rice flour. The bands at 1420–1330 cm^−1^ are attributed to the C–H bending vibrations of –CH_2_ due to the cis-distributed alkene groups, and the bands at 1300–1000 cm^−1^ are attributed to the CH_3_ bending vibrations, C–OH stretching and bending vibrations, and skeletal vibrations of C–O–C, which represent the presence of glycosidic linkages. The specific bands at 1153 to 1000 cm^−1^ indicate C–O–H bending and C=O stretching vibrations due to the presence of amylose and amylopectin polymers. Among the Karnataka rice cultivars, Kaveri, Salem Sanna, and Bili Muguthi showed differences at this position. However, in the Assam rice cultivars, Pare, Basmathi, and Kutkuti Sali were shown to have variations at the same peak positions with varied intensities. Changes in the intensity and shape of the peaks in this region may indicate variations in starch composition, degree of polymerization, and molecular interactions within the starch matrix of the different rice cultivars. Furthermore, it also reflects the functional parameters of rice grains, such as the tendency to gelatinize, retrograde, and digestible starch. The peaks beyond 930 cm^−1^ represent the hydrophilicity of rice flour, where the variations in the FTIR peak intensities among different rice cultivars indicate differences in water absorption capacity, swelling behavior, and moisture retention. Furthermore, the skeletal mode vibrations of the α-1,4 glycosidic linkages and glucopyranose ring structures are illustrated by the bands at 526–764 cm^−1^. The highly ordered structure of starch polysaccharides can be quantified using the absorbance ratio R (1047/1022) **(**Table 2). The values ranged from 1.0 to 1.25 for the Karnataka rice cultivar and from 1.0–1.14 for the Assam rice cultivar. The R values are proportional to the degree of the ordered structure the highly intense peaks at 1047 and 1022 cm^−1^ indicate the highly ordered amorphous and crystalline domains of starch (Fig. 2c,d). Overall spectral characteristics aid in the assessment of rice grain quality and provide valuable insights into the structural features and properties of starch, which are essential for various applications in food science, nutrition, and agriculture.

**Table 1:** Table showing FTIR band assignments for the rice cultivars:

Wavenumber (cm^−1^)	Band assignments	Prediction	References
3550-3200^b^	Stretching vibration of O−H	Polysaccharides	^43^
2927-2935^w^	CH_2_ asymmetric or symmetric stretch	Mainly unsaturated lipids and a small contribution from proteins, carbohydrates, nucleic acids	^44^
1628-1650^m^	C=O stretch (1652 cm^−1^) Cis C=C (1654 cm^−1^) H–O–H bending vibrations	Protein Bound water molecule	^44,45^
1420-1330^w^	C–H Bending vibrations of –CH_2_	Cis distributed alkenes	^46,47^
1300-1000^w^	CH_3_ bending vibrations, C–OH stretching and bending vibrations, skeletal vibrations of C–O–C	Lipids and proteins Presence of glycosidic linkages	^48,46,47^
1158^s^	C–O stretching of C–OH bonds	Starch	^49^
1080^s^	C–O–H bending and C–C stretching of C–O–C	Polymer chain	^46^
1048	Characteristic band of crystalline region of starch	Amylopectin chains	^50^
1020.94^s^	Characteristic to region amorphous, C–O of C–O–C	Amylose chains	^51,50^
930	Skeletal mode vibrations of C–O–C and Intramolecular bonding of –OH group at C–6 position in native starch	Presence of strong glycosidic linkages and indicates the hydrophilicity of starch	^52,47^
526–764 cm^−1^	Fingerprint region	Glucopyranose ring of starch	^53^

b-broad, w-weak, s-strong, m-medium.

**Table 2:** Table showing R1047/1022 for different indica rice cultivars:

Karnataka rice cultivars		Assam Rice cultivars	
Name of the rice cultivars	R 1047/1022	Name of the rice cultivars	R 1047/1022
Bananthi akki	1.01	Til bora	1.00
Bili muguthi	1.18	Basmathi	1.28
Betta sanna	1.10	kunkuruvai	1.10
Gandha Sale	1.00	Neuli	1.12
Kaveri	1.25	Jhum beji	1.14
Kempu Rajamudi	1.00	Aad bau	1.05
Kage Thali	1.15	Chakaw	1.10
Salem Sanna	1.23	Pare	1.20
Tanu	1.01	Thangam Champa	1.02
Bili rajamudi	1.02	Kutkuti sali	1.19

### Differential scanning calorimetry (DSC) of starch granules

**Fig. 3:**
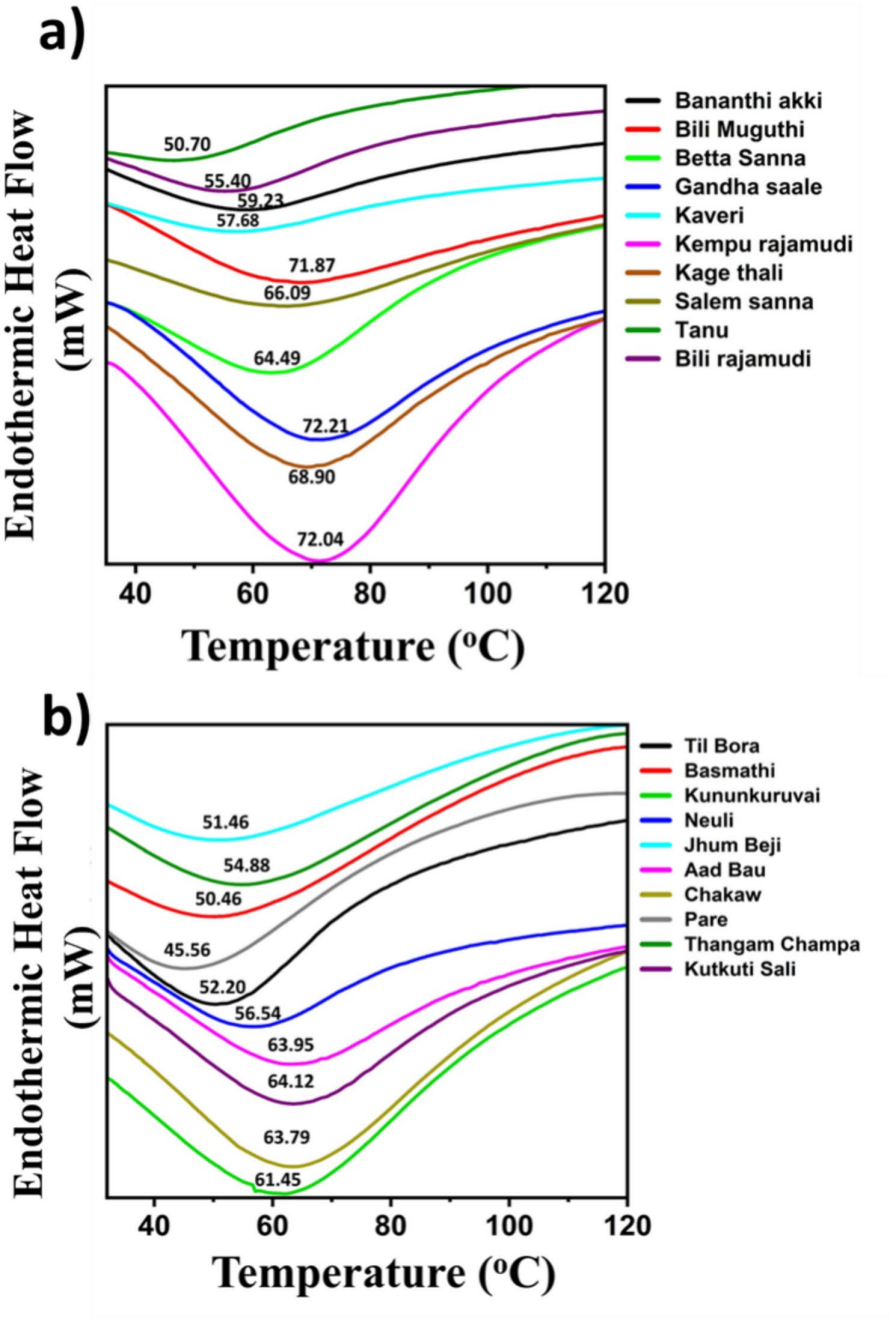
DSC thermograms depicting the peak gelatinization temperatures of (**a**) Karnataka and (**b**) Assam rice cultivars.

The thermal properties of the rice flour were determined using DSC. The onset (T_o_), endset (T_c_), and peak gelatinization (T_p_) temperatures varied significantly among the Karnataka and Assam rice cultivars. The T_o_, T_p_ and T_c_ ranged from 30.02–36.25 °C, 50.70–72.21 °C, and 68.12–88.87 °C, respectively, for the Karnataka rice cultivars. The temperatures of the Assam rice cultivars ranged from 30.01–38.20 °C, 45.56–64.12 °C, and 68.12–83.68 °C. A high gelatinization peak temperature was observed for Kempu Rajamudi (72.04 °C) in Karnataka and Kutkuti Sali (64.12 °C) in Assam (Fig. 3). The onset temperatures for both the Karnataka and Assam rice cultivars were lower than those for T_p_ and T_c_. This is because the initial swelling of starch granules does not require a high temperature, and hence, the onset temperatures are lower than the peak and endset temperatures. The gelatinization peak temperature increased in all the rice varieties due to the melting of the crystalline region (amylopectin) as gelatinization progressed. The endset temperature denotes the complete melting of crystalline regions (amylopectin) hence, the more highly ordered the amylopectin region is, the greater the endset temperature and gelatinization enthalpy^20^ (Table 3). The high gelatinization peak temperature in the Kempu Rajamudi and Kutkuti Sali rice varieties indicates the need for more heat for the gelatinization of starch granules. The predominant factors to be considered for the analysis of these variations are the amylopectin fine structure and its degree of branching. Crystalline amylopectin is also the major determinant of the variation in the gelatinization enthalpy. The disentanglement of double helices also illustrates the higher gelatinization enthalpy for the rice flours. The samples considered in this study showed a weak negative correlation, which was not significant between the amylose content and the gelatinization peak temperature however, there are various other studies that support the negative relationship between the amylose content and gelatinization peak temperature^11,21^ In addition, the differences in the total starch content, protein content, lipid content, and moisture content among the rice flours can also be attributed to variations in the transition temperature. The formation of amylose–lipid complexes, starch-bound proteins, and fiber content due to the presence of cell walls retards gelatinization, thereby increasing the gelatinization enthalpy^22^. Hence, in regard to the gelatinization of whole grain cereals, the interplay of all these factors justifies the variations in the gelatinization temperature, cooking time estimation, water uptake, and certain sensory attributes of the cooked rice varieties.

**Table 3:** In vitro digestion kinetics parameters for different rice cultivars of Assam and Karnataka.

Sl. No	Rice varieties	AUC	HI	GI	C∞	k (min^−1^)
Assam rice cultivars						
1	Neuli	485.62	96.47 ± 0.02^a^	92.67 ± 0.46^a^	91.21 ± 0.28^a^	0.063 ± 0.001^a^
2	Thangam Champa	450.05	89.40 ± 0.11^bx,e^	88.79 ± 1.34^b^	82.08 ± 2.35^c^	0.11 ± 0.064^d^
3	Til Bora	438.28	87.06 ± 0.18 ^bx^	87.51 ± 2.39^b^	79.71 ± 0.67^b^	0.056 ± 0^b^
4	Kununkuruvai	427.13	84.85 ± 0.14^d^	86.29 ± 1.05^b^	79.60 ± 0.91^b^	0.064 ± 0.006^a^
5	Jhum Beji	439.37	87.28 ± 0.04^b^	87.63 ± 2.48^b^	81.38 ± 0.78^bc^	0.081 ± 0.008^d^
6	Chakaw	440.43	87.49 ± 0.09^b^	87.75 ± 2.42^b^	83.70 ± 0.76^c^	0.056 ± 0.003^b^
7	Aad Bau	396.3	78.72 ± 0.13f.	82.93 ± 0.81^d^	77.01 ± 0.48^b^	0.046 ± 0.001^bc^
8	Kutkuti Sali	384.91	76.46 ± 0.22f.	81.69 ± 2.20^d^	73.25 ± 1.17^b^	0.049 ± 0.004^c^
9	Basmathi	355.18	70.55 ± 0.24^c^	78.45 ± 0.89^c^	68.18 ± 0.96^d^	0.052 ± 0.004^b^
10	Pare	358.88	71.29 ± 0.27^c^	78.85 ± 0.58^c^	67.68 ± 0.55^d^	0.058 ± 0.003^b^
Karnataka rice cultivars						
1	Bananthi akki	392.63	77.99 ± 1.23f.	82.53 ± 1.01^d^	82.22 ± 2.36^a^	0.029 ± 0.005^a^
2	Gandha Saale	379.59	75.4 ± 1.33^d^	81.11 ± 1.82^d^	82.26 ± 2.47^a^	0.024 ± 0.003^a^
3	Bili Rajamudi	366.83	72.87 ± 1.18^a^	79.72 ± 0.81^b^	71.01 ± 2.31^b^	0.045 ± 0.008^b^
4	Betta Sanna	362.85	72.08 ± 1.18^a^	79.28 ± 0.50^b^	71.18 ± 2.07^b^	0.042 ± 0.006^b^
5	Salem Sanna	357.79	71.07 ± 0.88^a^	78.73 ± 0.37^c^	69.64 ± 1.80^b^	0.043 ± 0.006^b^
6	Tanu	355.78	70.67 ± 0.76^b^	78.51 ± 0.75^c^	67.87 ± 2.43^c^	0.049 ± 0.011^b^
7	Bili Muguthi	356.14	70.75 ± 0.93^b^	78.55 ± 0.80^c^	73.66 ± 2.88^d^	0.03 ± 0.006^a^
8	Kempu Rajamudi	357.7	71.06 ± 0.67^a^	78.72 ± 0.68^c^	74.75 ± 2.40^d^	0.028 ± 0.005^a^
9	Kage Thali	343.155	68.17 ± 0.11^c^	77.14 ± 0.78^e^	69.47 ± 2.11^b^	0.033 ± 0.006^a^
10	Kaveri	362.98	72.1 ± 1.16^a^	79.30 ± 0.39^b^	74.29 ± 2.62^d^	0.031 ± 0.006^a^
	Fresh Bread	503.37	100 ± 0.91^e^	94.61 ± 0.77^a^	93.81 ± 2.21^a^	0.074 ± 0.017^c^

*AUC- area under the starch hydrolysis curve HI- hydrolysis index GI- glycemic index C∞—theoretical value of digested starch at the equilibrium point of starch hydrolysis *k* – rate constant for the starch hydrolysis reaction. All values are expressed as the mean of duplicate samples and the standard deviation with *p* < 0.05. Values with different letters in the same column are significantly different at* p* < 0.05.

### Determination of amylose content in the rice varieties

**Fig. 4:**
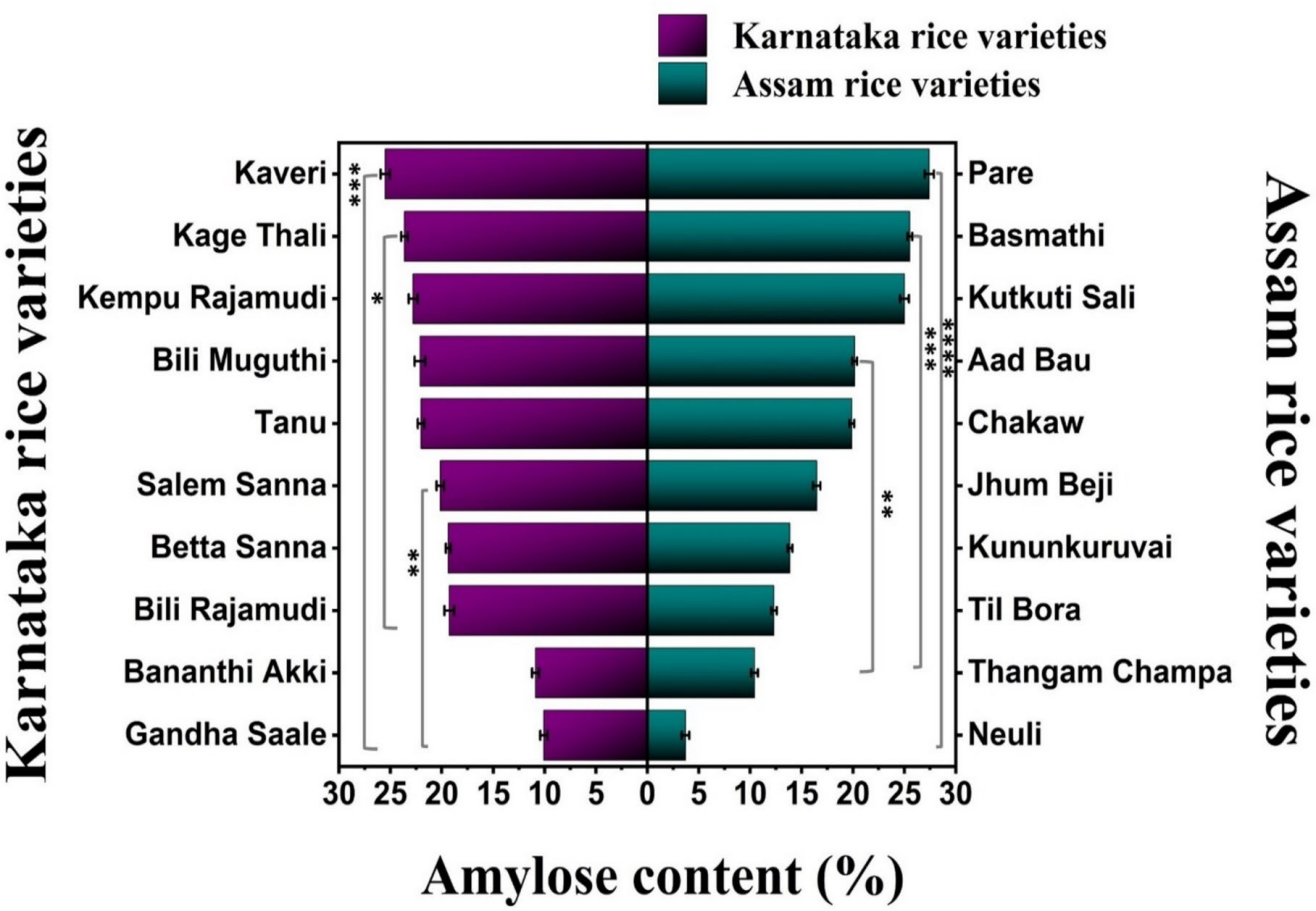
Horizontal bar graph depicting the amylose content of the Karnataka and Assam rice cultivars (**** *p* = 0.009, *** p = 0.014, ** p = 0.031).

The amylose content is an important parameter that determines the cooking and eating qualities of rice. The amylose content also determines the suitability of starch for industrial use. The amylose contents of the rice cultivars considered in this study significantly differed (*p* < 0.05). The rice cultivars are classified as very low (2–12%), low (12–20%), intermediate (20–25%), or high-amylose (> 25%) rice cultivars based on their amylose content. Among the Karnataka rice varieties, Kaveri had the maximum amylose content (25.50%), and Gandha Saale had the minimum amylose content (10.06%), whereas among the Assam rice varieties, Basmathi had the maximum amylose content (25.52%), and Neuli had the minimum amylose content (3.70%) (Fig. 4). The significant variations in the amylose content of rice cultivars are attributed to various genetic factors, degrees of milling, nitrogen fertilizers^23^, environmental factors^24^, etc. Temperature during the ripening of rice grains plays an important role in the development of starch molecular structure^25,26^. Since both regions have diverse climatic conditions in terms of temperature and rainfall, the variations in the amylose content can be attributed to these factors. Furthermore, macrolevel starch structures are also impacted by salinity and water-related stressors^27^. Since some of the enzymes responsible for starch biosynthesis are heat labile, atmospheric temperatures greater than or less than optimal can have a significant impact on their function during starch synthesis at the molecular level, i.e., the formation of amylose and amylopectin chains and their branching^28^. The use of more nitrogen and phosphorus fertilizers results in lower amylose levels in certain rice cultivars^23^.

### Determination of the swelling power of the rice varieties

**Fig. 5:**
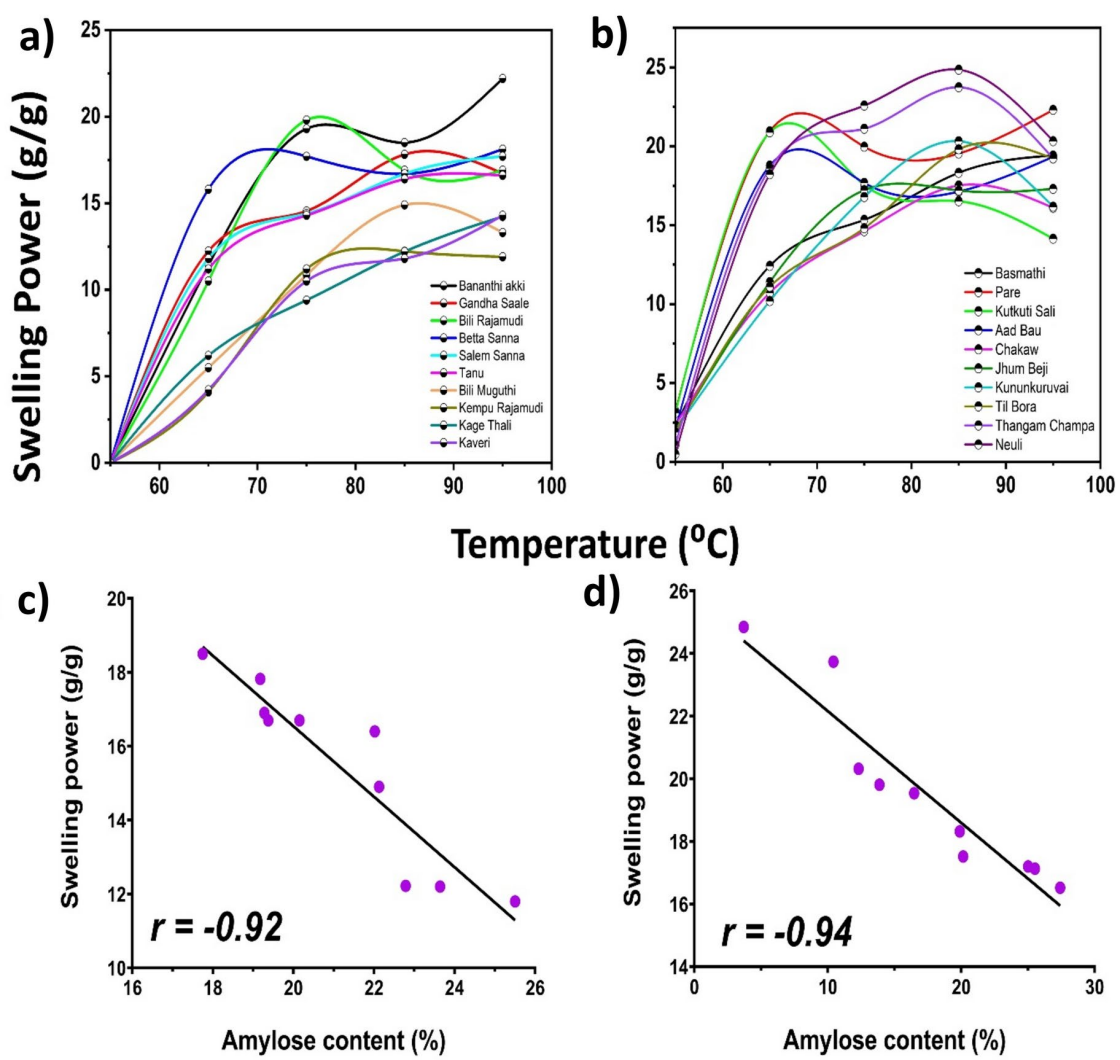
Graph depicting the swelling power of (**a**) Karnataka; (**b**) Assam rice cultivars relationship between swelling power and amylose content of (**c**) Karnataka; (**d**) Assam rice cultivars.

The swelling power is the ratio of wet sediment from rice flour to dry sediment. It determines the water holding capacity of rice flour and hence determines its applicability in various food industries. The swelling power of the rice starches ranged from 12.42 to 24.85 g/g for the Assam rice cultivars, whereas for the Karnataka rice cultivars, it ranged from 4.1 to 22.40 g/g over the temperature range of 55°–95 °C (Fig. 5). The Neuli and Thangam Champa rice varieties of Assam and the Bananthi Akki and Gandhasaale rice varieties of Karnataka showed greater swelling than the other rice cultivars. This could be due to their low amylose content. Additionally, both the Assam and Karnataka rice varieties with high amylose contents showed the least swelling. Furthermore, there was a strong negative correlation (*r* = − 0.9) between the amylose content and the swelling power of the rice cultivars, irrespective of their region of cultivation. In high-amylose rice cultivars, amylose is a single coiled helix that precludes the binding of water molecules, and the compact structure of amylose makes the starch rigid, which resists the absorption of water molecules into it and thus reduces its swelling power. In low-amylose rice cultivars such as Neuli, Thangam Champa, Bananthi Akki, and Gandhasaale, the amylose chains are easily disorganized upon cooking, and they interfere with the highly ordered, crystalline amylopectin region as cooking proceeds, leading to the forfeiting of its structure. Amylopectin is another fundamental component of starch, and its fine structure and molecular weight play important roles in the swelling process^28^ Apart from these two factors, the presence of nonstarch components such as proteins and lipids in whole-grain cereals results in the formation of starch-lipid and starch-protein complexes due to their interactions with starch. The formation of such complexes precludes swelling. In addition, other nonstarch components, such as dietary fiber and phenolics, can also contribute to the reduced swelling power of whole-grain rice flour^29^.

### Determination of resistant starch (RS), nonresistant starch (NRS), and total starch (TS)

**Fig. 6:**
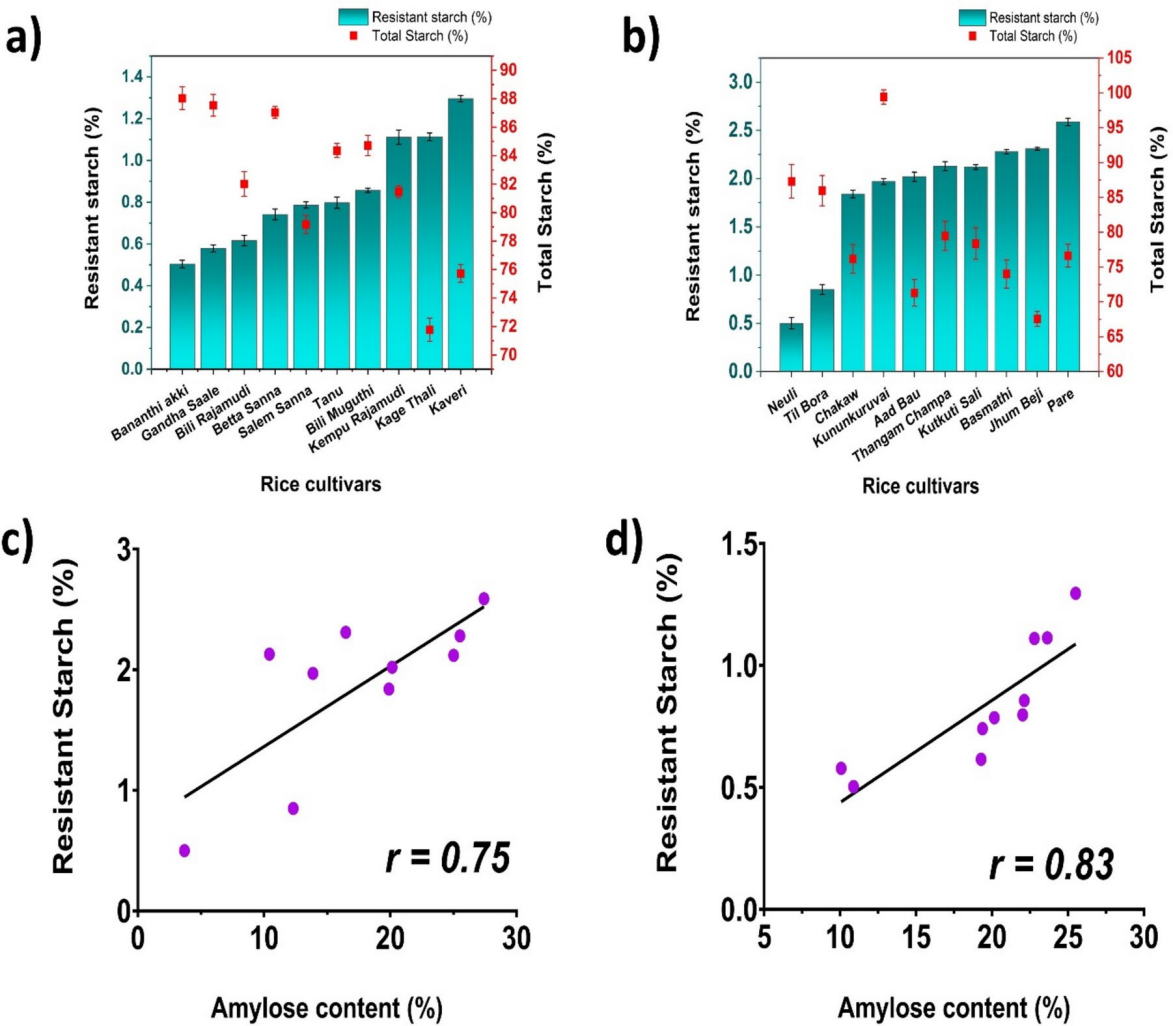
Graph depicting the total starch and resistant starch of (**a**) Karnataka; (**b**) Assam rice cultivars relationship between resistant starch and amylose content of (**c**) Karnataka; (**d**) Assam rice cultivars.

The rice varieties Karnataka and Assam showed a wide range of differences in their RS, NRS, and TS contents. In the case of the Karnataka rice variety, the highest RS value was obtained for Kaveri (1.296%), and the lowest RS value was obtained for Bananthi Akki (0.503%), whereas in the Assam rice variety, the highest RS was present in Pare (2.59%), and the lowest RS was observed in Neuli (0.50%). The RS of the Karnataka rice varieties ranged from 0.2–1.3%, whereas that of the Assam rice varieties ranged from 0.5–2.6%. The TS content varied from 72–88% for the Karnataka varieties and 67–98% for the Assam rice varieties (Fig. 6). The significant variation (*p* < 0.05) in the RS and TS contents can be attributed to external factors during the growth of plants, such as stress, altered climatic conditions, and fertilizer use. These external factors greatly contribute to the altered enzymatic activity during starch biosynthesis. Although raw and processed rice contain appreciable amounts of RS, the amount of RS differs according to the botanical origin, degree of processing, and genetic mutation of the RS genes. Although the samples considered in this study were from the same botanical source, the percentages of RS in the rice cultivars of Assam were greater than those in the Karnataka rice cultivars. The low-amylose rice cultivars from Karnataka and Assam both exhibited a lower RS content than did the high-amylose rice cultivars thus, there was a positive correlation between the amylose content and the RS in both the Karnataka and Assam rice cultivars, indicating that amylose chains are responsible for the presence of RS in different rice cultivars. This difference may be due to the arrangement of the amylose chains inside the starch granules. The presence of more amylose chains reduces the surface area available for the amylases to digest the starch granules. However, because amylopectin is largely branched, it provides a larger surface area and makes the starch granules least resistant to digestion. The primary barrier to amylase access is the single helix structure of amylose. Additionally, the tightly coiled amylose framework shields the internal hydrogen bonds of starch, preventing amylase penetration^30^. The structural features of starch granules also contribute to the ‘resistant’ nature of starch because, during the RS determination, the use of different enzymes follows different mechanisms of action with varied internal and external structural features of starch^20^. Additionally, the methodology used for RS quantification, direct methods^31^ and indirect methods^32,33^ has been shown to significantly affect the measurement of RS.

### Determination of the in vitro glycemic indices of the rice varieties

**Fig. 7:**
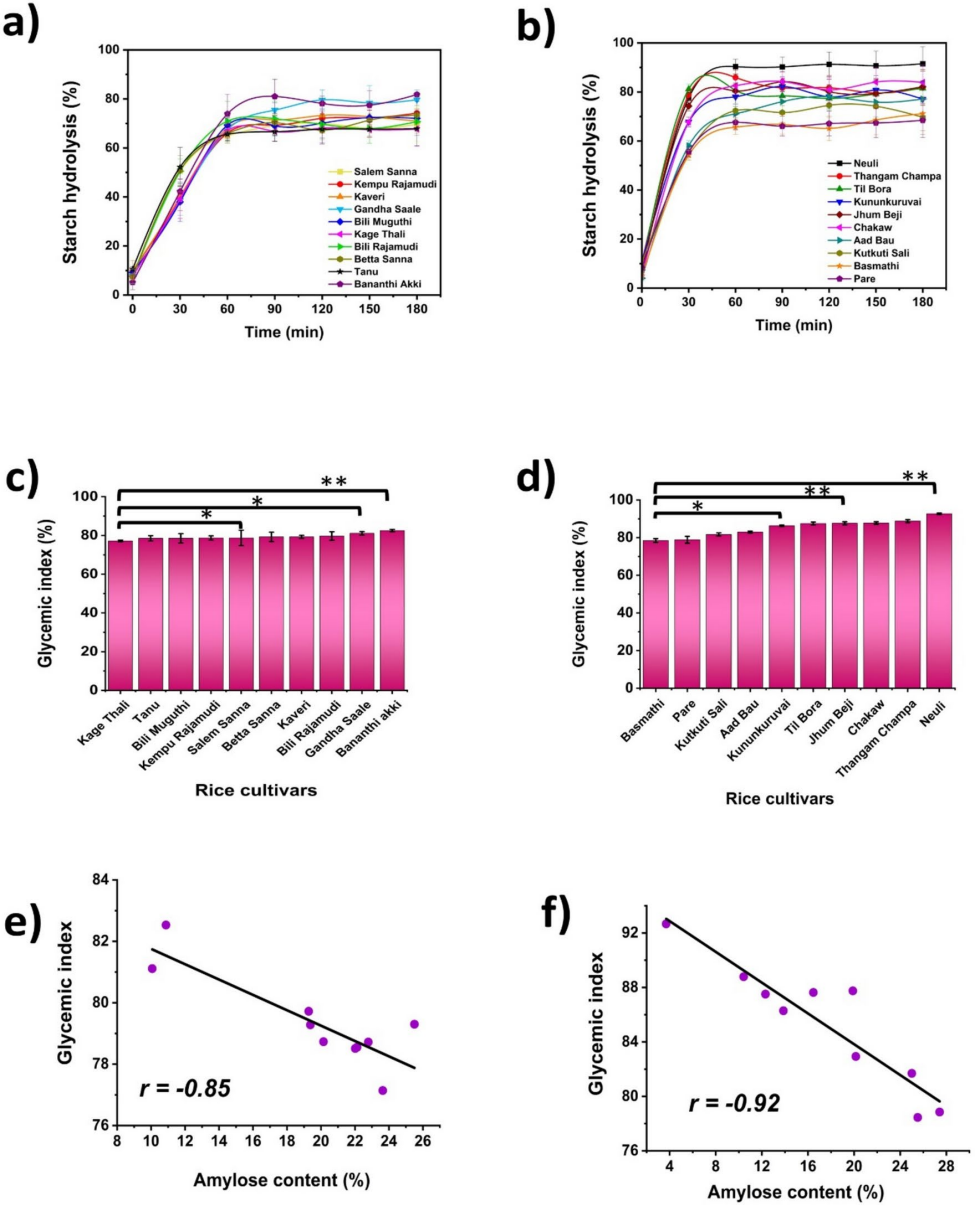
Graphical representation of in vitro enzymatic kinetics showing starch hydrolysis and estimated glycemic index values of (**a**) & (**c**) Karnataka, (**b**) & (**d**) Assam rice cultivars (* & ** *p* = 0.049 & 0.031, respectively). Graphs depicting the relationship between the amylose content and glycemic index of (**e**) Karnataka and (**f**) Assam rice varieties through Pearson correlation analysis.

Among the Karnataka rice cultivars, Bananthi Akki (82.53 ± 1.01) and Kage Thali (77.14 ± 0.78) had the highest and lowest eGIs, respectively. However, among the Assam rice varieties, the most common GI rice cultivar was Neuli (92.67 ± 0.46), and the least common eGI rice variety was Pare (78.85 ± 0.58), with a significant difference (*p* < 0.05) (Fig. 7). Regardless of the region where these plants are grown, the high-amylose rice cultivars exhibited less starch hydrolysis and lower eGI s than did the intermediate- and low-amylose rice cultivars. Similar findings have been reported by various authors, which supports the effect of high amylose content on reducing the digestibility of rice^34^. Usually, food is classified into high-GI rice cultivars (GI ≥ 70), medium-GI rice cultivars (GI 56–69), and low-GI rice cultivars (GI ≤ 55)^35^. In our study, all the rice cultivars were classified as high-GI rice cultivars (GI ≥ 70) based on their estimated GI values. Although the rice cultivars considered in our study were of the same botanical origin and had high GI values, there was significant (*p* < 0.05) variation in the HI and estimated GI of the Assam rice cultivars compared to the HI and estimated GI of the Karnataka rice varieties. The primary reason for this difference is the ratio of amylose to amylopectin in different rice cultivars. The molecular structure of these fundamental units plays an important role in determining starch digestibility. Amylose is always a single and coiled helix, and its ordered structure is well packed with strong hydrogen bonds in the starch interior. Thus, the amylose content of rice cultivars is directly proportional to the density of internal hydrogen bonds. This tightly packed configuration of amylose resists the penetration of enzymes into the starch interior and denies the accessibility of α-1,4 linkages^36,37^ This explains the decreased enzymatic susceptibility of high-amylose cultivars to α-amylases and thus the negative correlation between the GI and amylose content^38^. Furthermore, we observed the same trend in determining the resistant starch content, which clearly explains the role of high amylose content in reducing digestibility by increasing the resistant starch content (Fig. 6). However, few rice cultivars of Karnataka do not follow the above-discussed rule, and their deviation from this rule resulted in *a* Pearson correlation coefficient of − 0.85. This significant variation may be due to the different structural/morphological features of the starch granules. The presence of pores on the external surface of starch granules sometimes extends directly to the hilum region of the starch. The presence of such pores is a unique feature of A-type cereal starches^39^ and facilitates the direct diffusion of amylases to the core of starch granules. Once the enzyme achieves this, the resistance to enzymatic activity is dependent on the local molecular organization of the amorphous amylose and crystalline amylopectin of the starch polymers. This also explains the reduced rate constant ‘*k*’ in the Karnataka rice cultivar compared to the Assam rice cultivar. In addition, plants are exposed to a wide range of stresses, such as cold stress, drought stress, low-light stress, carbon dioxide stress, and salinity stress, due to climatic changes, which results in diverse and complicated responses with respect to the enzyme activities involved in starch biosynthesis^40–42^. Alterations in starch biosynthesis directly affect the granular structure of starch and thereby modify its digestibility.

### Statistical analysis

Pearson correlation analysis revealed a negative correlation among the amylose content, resistant starch content, and glycemic index of the rice cultivars. The Pearson correlation coefficients were r = − 0.85 and − 0.92 for the GI vs. amylose of the Karnataka and Assam rice cultivars, respectively. Similarly, the Pearson correlation coefficients were r = − 0.8 and − 0.6 for GI v/s RS, respectively. Furthermore, the swelling power was negatively correlated with the amylose content of both the Karnataka and Assam rice cultivars, with Pearson correlation coefficients of − 0.92 and − 0.94, respectively. However, the amylose content was positively correlated with the resistant starch content of both the Karnataka and Assam rice cultivars, with Pearson correlation coefficients of 0.75 and 0.83, respectively.

## Conclusion

In our study, we investigated the factors affecting the digestibility of *Indica* rice cultivars of Karnataka and Assam origin. Through in vitro digestion experiments, we examined the differences in digestion rates and patterns among the rice cultivars. This finding sheds light on their potential to increase postmeal blood sugar, and hence, all the rice cultivars are classified as high glycemic index rice cultivars. Although the samples considered in this study were from the same botanical source (*O. sativa indica*), the digestibility-determining characteristics of rice, such as resistant starch, total starch, most predominantly starch hydrolysis, and glycemic index, varied significantly. These variations are mainly attributed to the nature of the building blocks of polysaccharides present in these rice cultivars, i.e., the amylose and amylopectin of starch. The major determinant of rice digestibility is the molecular structure of the starch content, in which the ratio of amylose to amylopectin plays a vital role. Although all the rice varieties considered in our study were categorized as high-GI rice varieties, the slight variations in the GI values may be due to differences in the amylopectin chain length, degree of polymerization, surface-bound proteins, and formation of amylose–lipid complexes during the cooking process. DSC thermograms revealed variations in the gelatinization parameters, and the wide range of onset, peak, endset, and gelatinization enthalpies confirmed the altered structural framework of amylopectin among the rice cultivars. The limitation of our study is, In vitro tests cannot perfectly replicate the complex processes of human digestion and absorption. Factors such as the rate of gastric emptying, the activity of digestive enzymes, and the absorption of glucose in the intestines can all influence the GI of a food in ways that may not be accurately captured in an in vitro model. while in vitro GI testing can provide useful information about the potential glycemic impact of different foods, the results should be interpreted with caution due to the potential limitations of the methodology. Further research is needed to standardize in vitro GI testing protocols and to better understand how in vitro GI values relate to in vivo GI values and to the actual glycemic responses of human consumers. The findings of our study demonstrate the nutritional qualities of traditional rice consumed by the local populations of Karnataka and Assam, which is helpful for health-conscious consumers. Furthermore, the rice variety with the best grain characteristics is the most significant predictor of market grade and end use attributes. Thus, traditional rice types can be combined with white rice varieties to produce novel rice and rice starch-based food items such as rice noodles and rice cakes. This information will also aid in improving the nutritional well-being of the world’s growing population and combating lifestyle disorders associated with the metabolism of dietary carbohydrates and will aid in increasing the competitiveness of the global market and promoting the cultivation of traditional rice varieties.

## Data Availability

The authors declare that all the data supporting the findings of this study are available within the paper. Further data supporting the findings of this study are available from the corresponding author upon request.
